# Morphine Protects Spinal Cord Astrocytes from Glutamate-Induced Apoptosis via Reducing Endoplasmic Reticulum Stress

**DOI:** 10.3390/ijms17101523

**Published:** 2016-10-24

**Authors:** Chao Zhang, Chendan Wang, Jianbo Ren, Xiangjie Guo, Keming Yun

**Affiliations:** 1 Department of Forensic Medicine, Shanxi Medical University, 56 South Xinjian Road, Taiyuan 030001, China; zc_sxmu88@163.com (C.Z.); j_happy317@126.com (J.R.); xiangjieguo1980@yeah.net (X.G.); 2 Department of Nephrology, People’s Hospital of Shanxi Province, 29 Shuang-ta Street, Taiyuan 030012, China; cd_wang138@163.com

**Keywords:** astrocytes, glutamate, morphine, apoptosis, endoplasmic reticulum

## Abstract

Glutamate is not only a neurotransmitter but also an important neurotoxin in central nervous system (CNS). Chronic elevation of glutamate induces both neuronal and glial cell apoptosis. However, its effect on astrocytes is complex and still remains unclear. In this study, we investigated whether morphine, a common opioid ligand, could affect glutamate-induced apoptosis in astrocytes. Primary cultured astrocytes were incubated with glutamate in the presence/absence of morphine. It was found that morphine could reduce glutamate-induced apoptosis of astrocytes. Furthermore, glutamate activated Ca^2+^ release, thereby inducing endoplasmic reticulum (ER) stress in astrocytes, while morphine attenuated this deleterious effect. Using siRNA to reduce the expression of κ-opioid receptor, morphine could not effectively inhibit glutamate-stimulated Ca^2+^ release in astrocytes, the protective effect of morphine on glutamate-injured astrocytes was also suppressed. These results suggested that morphine could protect astrocytes from glutamate-induced apoptosis via reducing Ca^2+^ overload and ER stress pathways. In conclusion, this study indicated that excitotoxicity participated in the glutamate mediated apoptosis in astrocytes, while morphine attenuated this deleterious effect via regulating Ca^2+^ release and ER stress.

## 1. Introduction

Astrocytes are ubiquitous in central nervous system (CNS) and mediate multiple functions via releasing nutrients, growth factors, cytokines, and several neurotransmitters, thereby interacting with neurons and other glial cells [1]. Meanwhile, astrocytes could support the survival of neurons both in vivo [2] and in vitro through attenuating toxic effects [3]. In some pathological conditions, external stimuli could induce sustained release of some neurotransmitters in neuronal and glial cells. In which, glutamate, an important excitatory neurotransmitter, could activate calcium signals and mediates interaction between neuronal and glial cells [4]. However, chronic elevation of extracellular glutamate levels is known to injure neuronal cells and lead to brain disorders like epilepsy and Parkinson’s disease [5]. During early stage of glutamate elevation, glutamate receptor in astrocytes could be activated preferentially to minimize the deleterious effect of glutamate on neuronal cells [6,7]. However, prolonged activation by glutamate induces apoptosis in astrocytes, subsequently causing CNS injury [7]. Thus, astrocytes act as a defender in glutamate-induced neuronal cell apoptosis. Excitotoxicity and oxidative stress have been reported to participate in glutamate-induced cytotoxicity [8,9], but the precise underlying mechanism still needs further investigation.

Morphine is a well-known opioid ligand, which implicated in various different functions. Commonly, it acts as a powerful pain reliever via activating opioid receptor in the central nervous system (CNS) and peripheral nervous system [10]. The functions of morphine are mediated mainly through specific opioid receptor, μ, δ, and κ receptors in different regions and types of brain cells like neuronal and glial cells, thereby activating membrane G proteins to modulate adenylyl cyclase and intracellular concentration of Ca^2+^ ([Ca^2+^]_i_) [10,11]. Thus, morphine mediates multiple effects in CNS, leading to analgesia and other effects, including drug tolerance and dependence [12]. However, its underlying effects are complex and remain controversial. It was reported that morphine induced apoptosis in a wide variety of cells [13]. On the other hand, opioids, including morphine, were reported to inhibit some deleterious factors, such as glutamine, peroxynitrite, and nitric oxide (NO)-induced dysfunctions in both neuronal and glial cells [14,15].

Glutamate-induced excitotoxicity in CNS is mainly through regulating adenylate cyclase (AC) and phosphatidylinositol phospholipase C (PLC), thereby inducing chronic increased intracellular cAMP and Ca^2+^ level [8]. As morphine and other opioids could inhibit calcium release via activating opioid receptors, we speculated that morphine might reduce glutamate-induced excitotoxicity in CNS cells. The aim of this study was to investigate whether morphine could affect glutamate-induced apoptosis in primary cultured astrocytes.

## 2. Results

### 2.1. Morphine Protected Primary Cultured Spinal Cord Astrocytes from Glutamate-Induced Mitochondrial Apoptosis

**Figure 1:**
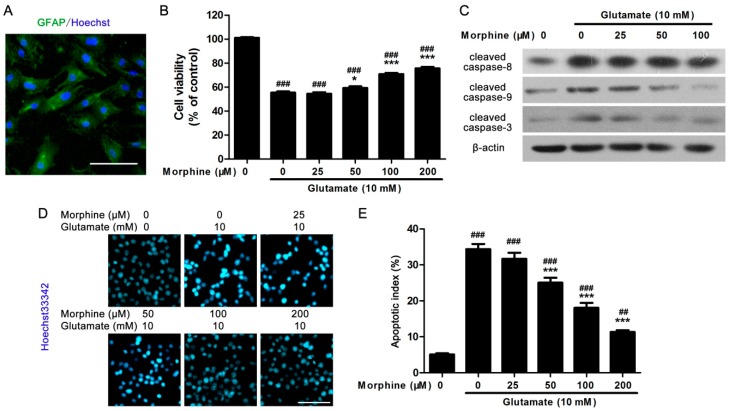
Morphine reduced glutamate-induced astrocytes apoptosis. (**A**) Following 14 days’ culture, astrocytes were stained by green fluorescence marked GFAP antibody, nuclei were stained with blue fluorescence by Hoechst 33342 dye. Scale bar = 50 μm; (**B**) following cells were treated with glutamate in the presence/absence of different doses of morphine for 24 h, MTT assay was performed to detect cell viability, * *p* < 0.05, *** *p* < 0.001 compared to glutamate only treated group, ^###^ *p* < 0.001 compared to morphine only treated group, *n* = 6; (**C**) following 24 h treatment, the cells were lysed by lysis buffer and the protein was extracted, then the expression of cleaved caspase-8, caspase-9, and caspase-3 were detected by western blot, β-actin was internal control, blots were representative of three independent experiments; (**D**) following 24 h treatment, the cells were fixed and stained with Hoechst 33342 dye, bright colored condensed nuclei indicated apoptotic cells. Scale bar = 50 μm and referred to all panels; (**E**) statistical analysis of Hoechst apoptotic staining, *** *p* < 0.001 compared to glutamate only treated group, ^##^ *p* < 0.01, ^###^ *p* < 0.001 compared to morphine only treated group, *n* = 3.

Primary cultured spinal cord astrocytes were isolated from newborn rats. As glial fibrillary acidic protein (GFAP) is specially expressed in the astrocytes, before experiment, GFAP antibody was used to identify the purity of astrocytes. Over 95% of cells were GFAP-positive (Figure 1A). It was found that after incubation with 10 mM glutamate for 24 h, cell viability of cultured astrocytes was significantly reduced compared to control astrocytes (Figure 1B). However, co-incubation with morphine dose-dependently prevented astrocytes from glutamate-induced cell death (Figure 1B), while morphine-treated-only cells had less change in cell viability up to 200 μM [16].

We further tested whether the effect of morphine on glutamate-induced decreasing in cell viability was related to apoptosis, some apoptotic markers were detected. It was found that glutamate increased the expression of representative apoptotic markers, including cleaved caspase-8, cleaved caspase-9, and cleaved caspase-3 in astrocytes. Meanwhile, although morphine affected less on elevated expression of cleaved caspase-8 under glutamate stimulation (Figure 1C), glutamate-increased cleaved caspase-9 and cleaved caspase-3 expression were both inhibited by morphine in a dose-dependent manner (Figure 1C), which strongly suggested that mitochondrial apoptosis could be involved in this process. Moreover, using Hoechst 33342 apoptotic detection kit, the effect of morphine on glutamate-induced astrocytes apoptosis was further investigated. Under Hoechst 33342 staining, apoptotic cells under fluorescence microscope would show brighter blue fluorescence with a high condensed chromatin and nuclei shrinkage. Consistent with above data, Hoechst 33342 assay also confirmed that morphine protected astrocytes from glutamate-induced apoptosis (Figure 1D,E).

### 2.2. Morphine Reduced Glutamate-Induced Ca^2+^ Release and Endoplasmic Reticulum (ER) Stress

**Figure 2:**
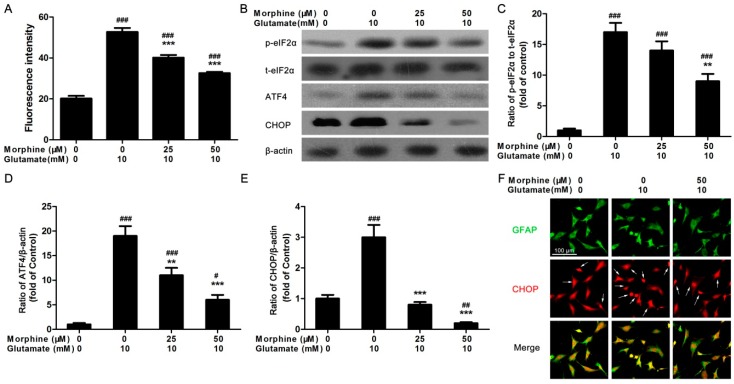
Morphine reduced glutamate-induced Ca^2+^ release and ER stress. (**A**) Acute intracellular calcium release was initiated by glutamate in astrocytes in the presence/absence of morphine. *** *p* < 0.001 compared to glutamate only treated group, ^###^ *p* < 0.001 compared to morphine only treated group, *n* = 6; (**B**) following 24 h treatment, the cells were lysed and the protein were extracted to detect the expression of phosphorylated eukaryotic initiation factor 2α (p-eIF2α), total eukaryotic initiation factor 2α (t-eIF2α), ATF4, and CHOP, β-actin was internal control, blots were representative of three independent experiments; The optical density of each band was analyzed as (**C**) the ratio of p-eIF2α to t-eIF2α, (**D**) ratio of ATF4/β-actin, (**E**) ratio of CHOP/β-actin, ** *p* < 0.01, *** *p* < 0.001 compared to glutamate only treated group, ^#^ *p* < 0.05, ^##^ *p* < 0.01, ^###^ *p* < 0.001 compared to morphine only treated group, *n* = 3; (**F**) following 24 h treatment, the cells were fixed and stained by green fluorescence-conjugated GFAP antibody and red fluorescence-conjugated CHOP antibody, white arrows indicated CHOP-positive cells, representative image was selected and merged. Scale bar = 100 μm and referred to all panels.

Glutamate-stimulated Ca^2+^ release was detected using fluo-4 dye. It was found that acute glutamate stimulation could increase intracellular concentration of Ca^2+^ ([Ca^2+^]_i_) to about 2.5-fold in astrocytes (Figure 2A). However, pre-incubation with morphine dose-dependently prevented glutamate-activated Ca^2+^ release (Figure 2A). It was reported that increased [Ca^2+^]_i_ might be associated with glutamate-induced excitotoxicity in astrocytes [17]. Therefore, we speculated that Ca^2+^ overload-induced ER stress might be involved in the detrimental effect of glutamate. To identify whether morphine affected this process, astrocytes were incubated with glutamate for 24 h in the absence or presence of different doses of morphine. Then the cells were lysed and total protein was extracted to determine the expression levels of proteins involved in ER stress. Prkr-like endoplasmic reticulum kinase (PERK) pathway was first examined. Phosphorylation of eukaryotic initiation factor 2α (eIF2α), the expression of activating transcription factor 4 (ATF4) and C/EBP homologous protein (CHOP) were analyzed by western blotting. We observed that glutamate caused an increased level of phosphorylation of eIF2α, expression of ATF4 and CHOP (Figure 2B–E). However, morphine prevented astrocytes from glutamate-induced increase in these ER stress markers in a dose-dependent manner (Figure 2B–E), suggesting a protective effect of morphine on cultured astrocytes. Consistent with the result of western blot, immunofluorescent data also indicated that morphine protected astrocytes from glutamate-increased CHOP expression (Figure 2F).

### 2.3. Inositol Requiring Kinase 1 (IRE1) Pathway Is Partially Involved in the Effect of Morphine on Glutamate-Treated Astrocytes

**Figure 3:**
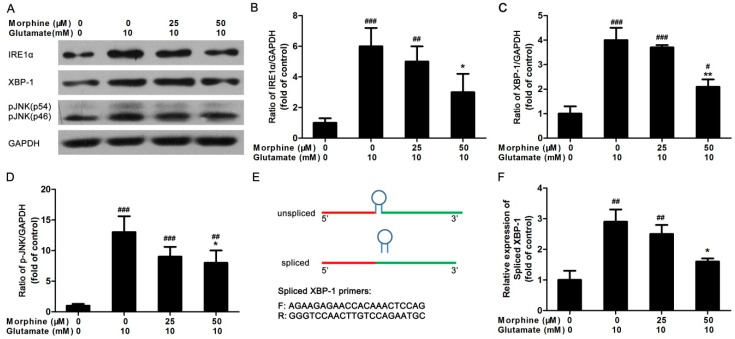
The effect of morphine on IRE1 pathway in glutamate-treated astrocytes. (**A**) Following 24 h treatment, the cells were lysed and the protein were extracted to detect the expression of IRE1α, XBP-1, and phosphorylated JNK (p-JNK), GAPDH was internal control, blots were representative of three independent experiments; The optical density of each band was analyzed as (**B**) ratio of IRE1α/GAPDH, (**C**) ratio of XBP-1/GAPDH, (**D**) ratio of p-JNK/GAPDH, * *p* < 0.05, ** *p* < 0.01, compared to glutamate only treated group, ^#^ *p* < 0.05, ^##^ *p* < 0.01, ^###^ *p* < 0.001 compared to morphine only treated group, *n* = 3; (**E**) the schematic of XBP-1 mRNA splicing and the sequence of primers for spliced XBP-1 mRNA used in experiment; (**F**) following 24 h treatment, the cells were lysed and total mRNA was extracted. Then real-time PCR was performed to detect the mRNA expression of spliced XBP-1. * *p* < 0.05 compared to glutamate only treated group, ^##^ *p* < 0.01 compared to morphine only treated group, *n* = 6.

We next measured the levels of key IRE1 pathway markers in ER stress, IRE1α, X-box binding protein 1 (XBP-1), and phosphorylation of c-Jun N-terminal kinase (JNK). After the cells were incubated with glutamate in the presence of different doses of morphine for 24 h, the expression of IRE1α, XBP-1, and phosphorylation of JNK were detected by western blot. As shown in Figure 3A, increased level of IRE1α, XBP-1, and phosphorylation of JNK were observed in glutamate-treated astrocytes compared to control cells. However, morphine co-incubation significantly suppressed the expression of proteins involved in IRE1 pathway in glutamate-treated astrocytes (Figure 3A–D).

Moreover, XBP-1 mRNA could be spliced to an active form by IRE1 via removal of 26 nucleotides from unspliced XBP-1 (Figure 3E). Using spliced XBP-1 primers, real-time PCR was performed to detect the mRNA expression level of spliced XBP-1. It was found that glutamate-induced increase in the mRNA expression of spliced XBP-1 was dose-dependently suppressed by morphine (Figure 3F). Although the alterations of proteins participating in the IRE1 pathway were not as strong as that in the PERK pathway, it could still inferred that IRE1 pathway was still partially involved in the effect of morphine on glutamate-induced apoptosis of astrocytes.

### 2.4. Knockdown κ-Receptor Suppressed the Protective Effect of Morphine on Glutamate-Stimulated Astrocytes

**Figure 4:**
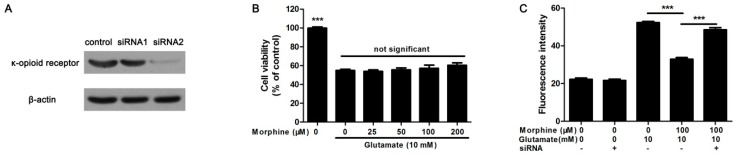

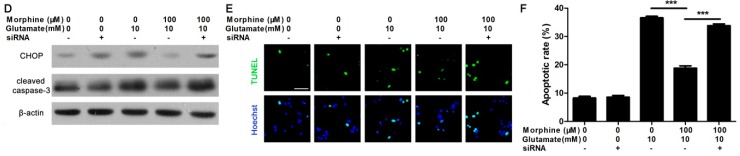
Knockdown κ-receptor reduces the protective effect of morphine on glutamate-treated astrocytes. (**A**) To detect the knockdown efficacy of two different siRNAs, the expression of κ-receptor was detected by western blot after the cells were treated with siRNAs, β-actin was internal control; (**B**) following 24 h treatment, cell viability in κ-opioid receptor knockdown astrocytes was determined by MTT assay, *** *p* < 0.001 compared to glutamate only treated group, *n* = 6; (**C**) glutamate-initiated calcium release in κ-opioid receptor knockdown astrocytes was determined by fluorescence microplate. *** *p* < 0.001 indicated significances, *n* = 6; (**D**) following 24 h treatment, the cells were lysed and the protein was extracted to detect the expression of CHOP and cleaved caspase-3, β-actin was internal control, blots were representative of three independent experiments; (**E**) following 24 h treatment, the cells were fixed and TUNEL staining was performed to detect apoptotic cells. Green fluorescence was TUNEL-positive nuclei, blue fluorescence was all nuclei stained by Hoechst 33342, scale bar = 50 μm and referred to all panels; (**F**) apoptotic rate was calculated as TUNEL-positive nuclei divided by total nuclei, *** *p* < 0.001 indicated significances, *n* = 15.

To investigate the effect mechanism of morphine on glutamate-induced astrocyte apoptosis, we studied whether the protective effect of morphine was through its κ-opioid receptor. Over 70% of κ-opioid receptor expression was silenced by siRNA2 (Figure 4A). It was found that, in κ-opioid receptor knockdown astrocytes, morphine affected cell viability less in glutamate-treated astrocytes (Figure 4B). Meanwhile, morphine could not inhibit glutamate-activated Ca^2+^ release in κ-opioid receptor knockdown astrocytes, either (Figure 4C). Consequently, in κ-opioid receptor knockdown astrocytes, morphine did not reduce glutamate-induced increase in CHOP and cleaved caspase-3 (Figure 4D). Consistent with these data, Hoechst 33342 staining showed that the protective effect of morphine on glutamate-treated astrocytes was suppressed in κ-opioid receptor knockdown cells (Figure 4E,F). Altogether, these data clearly indicated that the protective effect of morphine was specific dependent on its κ-opioid receptor.

## 3. Discussion

As the defender of CNS, astrocytes play an important role in the protection of CNS [18,19]. Impairment of astrocytes could injure water and metabolic support, transmitter uptake in CNS cells, thereby influencing neuronal survival [20,21]. Some deleterious factors—for example: sustained elevation of glutamate—could cause apoptosis in astrocytes, subsequently leading to CNS injury [22]. Similar to previous studies [22,23], after the cells were exposed to chronic treatment of glutamate, the cell viability of astrocytes reduced significantly, apoptotic detection also confirmed that chronic treatment of glutamate caused obvious apoptosis in astrocytes. Excitotoxicity was known to be one of the main causes of glutamate-induced apoptosis [24]. However, the detailed mechanisms were complex and still remain unknown. It was found that the expression of cleaved caspase-3, caspase-8, and caspase-9 increased significantly in chronic glutamate treated astrocytes, which suggested that mitochondrial apoptosis might be involved in this process. Thus, we speculated that after pro-apoptotic stimulation of glutamate, releasing of cytochrome c from mitochondria would cleave the pro-enzyme of caspase-8/caspase-9 into the active form, thereby activating caspase-3 to induce cell apoptosis [25,26]. However, while co-incubating morphine with glutamate in astrocytes, glutamate-induced apoptosis was significantly suppressed. As a powerful pain reliever, morphine could inhibit the conduction of neurotransmitters in CNS [27]. Thus, glutamate-induced excitotoxicity and apoptosis in astrocytes might be suppressed by morphine. It was also observed that morphine reduced glutamate-induced increase in the expression of cleaved caspase-3 and caspase-9. However, caspase-8 activation was affected less, suggesting that morphine mainly intervened in glutamate-induced mitochondrial apoptosis but not in death receptor pathway in astrocytes.

We further detected that whether or not the anti-apoptotic effect of morphine was through inhibiting of glutamate-induced excitotoxicity. Glutamate could activate Ca^2+^ release in multiple cell types including astrocytes to mediate signal transduction [28,29]. However, sustained elevation of Ca^2+^ release leads to excitotoxicity [30]. Morphine could activate opioid receptor in astrocytes to inhibit adenylyl cyclase, thereby reducing Ca^2+^ release [31]. Thus, it could be speculated that morphine intervenes in glutamate stimulated Ca^2+^ release. Using intracellular calcium mobilization assay, we found that glutamate significantly increased [Ca^2+^]_i_ in cultured astrocytes, while morphine dose-dependently suppressed this effect, suggesting that sustained elevation of Ca^2+^ release was involved in the excitotoxicity of glutamate. Interestingly, although 25 and 50 μM morphine obviously suppressed glutamate-induced acute Ca^2+^ release and chronic ER stress in astrocytes, under these dosages, especially 25 μM of morphine had less protective effect on cell survival in glutamate-treated cells. Not only excitotoxicity, but also other stress factors, like oxidative stress, are involved in glutamate-induced cell injury [9]. Therefore, it could be speculated that the protective effect of 25 μM of morphine was not enough to fully abolish glutamate-induced cell apoptosis.

Therefore, we mainly explored the effect of morphine on glutamate-induced ER stress in astrocytes. It was known that sustained elevation of [Ca^2+^]_i_ could induce some deleterious effect, for example, ER stress [17,32]. Ca^2+^ overload causes dysfunction in ER Ca^2+^ depletion, subsequently inducing accumulation of misfolded proteins in ER, this is termed unfolded protein response (UPR) [33]. During this process, some ER membrane proteins are activated, including IRE1, PERK, and activating transcription factor 6 (ATF6) [34]. These ER stress transducers could further activate CHOP and other pro-apoptotic factors, finally causing cell apoptosis [35,36]. Therefore, glutamate-induced apoptosis of astrocytes could be highly associated with Ca^2+^ overload caused ER stress. In this study, we investigated the effect of glutamate on ER stress in astrocytes. It was found that in glutamate-treated astrocytes, phosphorylation of eIF2α increased significantly compared to control cells. Consequently, activation of eIF2α enhanced the expression of ATF4, which induced pro-apoptotic transcription factor CHOP, finally activating the mitochondrial apoptotic pathway. However, using morphine to inhibit glutamate-induced sustained elevation of [Ca^2+^]_i_, the increased expressions of phosphorylated eIF2α, ATF4, and CHOP were all reduced, which contributed to the protective effect of morphine on astrocytes. In addition, we found another ER stress factor, IRE1 could be also activated by glutamate. In IRE1 pathway, IRE1 splices XBP-1 mRNA to active form that induces ER-associated protein degradation [37]. Activation of IRE1α resulted in splicing of XBP-1, thereby activating JNK, which also led to increased level of CHOP expression [38]. It was found that morphine inhibited glutamate-induced increase in the expression of IRE1α, moreover, the splicing of XBP-1 mRNA and phosphorylation of JNK were both reduced in morphine co-treated cells. In addition, another ER stress marker, ATF6 was affected less in this process [16], Therefore, ER stress, especially the PERK pathway, could be strongly involved in glutamate-induced astrocytes apoptosis, while morphine inhibit glutamate-induced Ca^2+^ overload, thereby contributing benefit for preventing ER stress and mitochondrial apoptosis in astrocytes.

Morphine plays a complex role in CNS, some studies showed that morphine could increase apoptosis in astrocytes [13,39], but it was also reported that morphine reduced astrocytes apoptosis under some deleterious factors [14,15]. Thus, the detailed effects of morphine on astrocytes and CNS still need further exploration. In this study, using siRNA, we knockdown the κ-opioid receptor on cultured astrocytes. As morphine generates inhibitory and analgesic effects mainly through κ-opioid receptors at the spinal level [40], we attempted to investigate the specificity of morphine on glutamate-induced apoptosis in spinal cord astrocytes. As shown in results, κ-opioid receptor knockdown did not affect glutamate-induced Ca^2+^ overload and ER stress associated apoptosis in astrocytes. However, the protective effects of morphine on glutamate-treated cells were obviously suppressed. It suggested that morphine specifically activated κ-opioid receptor, thereby reducing glutamate-caused Ca^2+^ overload and ER stress, to protect astrocytes from glutamate-induced apoptosis.

## 4. Materials and Methods

### 4.1. Cell Culture

Newborn (one day after birth) Sprague-Dawley rats were used in this experiment. Primary spinal cord astrocytes culture was performed as previous described [41]. Briefly, meninges were carefully removed, then spinal cords were dissected and dissociated in trypsin (0.25%) for 5 min at 37 °C. After centrifugation, the supernatant were removed and the cells were filtered by a 200 μm mesh sieve. Then the cells were seeded into a 6-well plate in DMEM (10% FBS, 100 IU/mL penicillin and 100 μg/mL streptomycin, GIBCO, Carlsbad, CA, USA) and cultured at 37 °C and 5% CO_2_ condition. All experiments were performed after 14 days culture to purify astrocytes. Over 95% of cells were GFAP-positive. Animal experiments were approved by the Animal Ethics Committee of Shanxi Medical University (2011004, January 2011).

### 4.2. Cell Viability Assay

After 14 days culture, astrocytes were seeded onto 96-well plates (15,000 cells per well) and left overnight for adherence. Then 10 mM glutamate and different concentration of morphine were added into culture medium for 24 h. Cell viability was determined by 3-(4,5-dimethyl-2-thiazolyl)-2,5-diphenyl-2-*H*-tetrazolium bromide (MTT) assay. MTT reagents (0.5 mg/mL) were added to each well for 4 h, then culture medium was removed and the dimethyl sulfoxide (DMSO) was added to dissolve the insoluble purple formazan product. The absorbance was measured by MK3 multiskan (Thermo Fisher, Waltham, MA, USA) at 490 nm.

### 4.3. Western Blot Analysis

After astrocytes were treated with 10 mM glutamate and different doses of morphine for 24 h, the cells were lysed by RIPA lysis buffer (Beyotime, Haimen, China) according to the protocol. Western blot was carried out as previously described with minor modification [42]. The primary antibodies used in experiments were: rabbit anti-cleaved caspase-8, rabbit anti-cleaved caspase-9, rabbit anti-cleaved caspase-3, rabbit anti-phosphor-eIF2α, rabbit anti-eIF2α, rabbit anti-ATF4, mouse anti-CHOP, rabbit anti-IRE1α, rabbit anti-XBP-1, rabbit anti-phosphor-JNK and rabbit anti-GAPDH antibodies (Cell Signaling Technology, Beverly, MA, USA), mouse anti-β-actin antibody (Sigma-Aldrich, St. Louis, MO, USA). Secondary antibodies were purchased from Jackson Laboratory (Sacramento, CA, USA). Each blot was repeated at least three times, the optical density of each band was measured by Image J software (National Institutes of Health, Bethesda, MD, USA).

### 4.4. Real-Time PCR Assay

Real-time PCR was used to detect the mRNA expression of spliced XBP-1. After astrocytes were treated with 10 mM glutamate and different doses of morphine for 24 h, the cells were lysed by TRIzol reagent (Takara, Shiga, Japan) according to the protocol. Total mRNA was reverse transcribed to synthesis cDNA by M-MLV kit (Invitrogen, Waltham, MA, USA). Then cDNA was amplified for 35 cycles in Light Cycler 480 (Roche Diagnostics, Indianapolis, IN, USA). The splicing of XBP-1 (the ratio of spliced XBP-1 to total XBP-1) was calculated by comparative *C*t, which was normalized by an internal standard (GAPDH mRNA). The primers used were: mouse total XBP-1: Forward: AGCTTTTACGGGAGAAAACTCAC; Reverse: CCTCTGGAACCTCGTCAGGA; mouse spliced XBP-1: Forward: AGAAGAGAACCACAAACTCCAG; Reverse: GGGTCCAACTTGTCCAGAATGC; mouse GAPDH: Forward: AATGGATTTGGACGCATTGGT; Reverse: TTTGCACTGGTACGTGTTGAT.

### 4.5. Hoechst 33342 Staining

For Hoechst 33342 staining, the cells were planted into 24-well plates on cover glass. After astrocytes were treated with 10 mM glutamate and different doses of morphine for 24 h, the cells were fixed in 4% paraformaldehyde for 2 h, followed by multiple washes with phosphate buffered saline (PBS), then Hoechst 33342 dye (Beyotime) was added into medium and incubated for 30 min. The cells were observed under fluorescent microscope (DP70, Olympus, Tokyo, Japan), 10 random fields from each group (triplicate wells) were selected to analysis apoptotic ratio.

### 4.6. Calcium Mobilization Assay

Cells were put into 96-well plates overnight. Then the culture medium was replaced with 2 μM fluo-4 dye (Invitrogen) and 2.5 mM probenecid (Sigma-Aldrich) in Hanks’ balanced salt solution (HBSS, Beyotime) for 1 h. After washes by HBSS, the cells were pre-incubated with different doses of morphine for 10 min, then 10 mM glutamate was added into wells to stimulate calcium release, which was quantified by the fluorescence absorption of fluo-4 dye using a fluorescent microplate reader (Flexstation, Molecular Devices, Sunnyvale, CA, USA).

### 4.7. Immunofluorescence

For double immunofluorescence of GFAP and CHOP, cells were planted on a cover glasses in 6-well plates (500,000 cells/well). After treatment with glutamate and morphine, the cells were fixed in 4% paraformaldehyde for 2 h and washed by PBS. Antigens were blocked in 1% bovine serum albumin (BSA) for 15 min at room temperature. Then monoclonal primary antibody rabbit anti-GFAP (dilution 1:100, Abcam, London, UK) was first added on the glasses and incubated at 4 °C overnight. Following multiple PBS washes, the cells were incubated with the AlexaFluor 488-conjugated goat anti-rabbit IgG (dilution 1:400, Invitrogen) in the dark for 2 h at room temperature. Then the cells were washed and stained by another primary antibody mouse anti-CHOP for 5 h at room temperature, followed by corresponding secondary antibody AlexFluor 545-conjugated goat anti-mouse IgG (dilution 1:400, Invitrogen). The cells were washed and mounted on glass slides. The images were captured by a fluorescent microscope (DP70, Olympus).

### 4.8. TUNEL Staining

Cells were cultured on cover glasses in 6-well plates. After treatment, cells were stained by an in situ cell death detection kit (Roche Diagnostics) according to its protocol. The apoptotic cells were stained with green fluorescence, all nuclei were counterstained by Hoechst 33342 dye. For statistics, 10 random sights in each well were selected, apoptotic ratio was calculated as the apoptotic cells divided by total cells.

### 4.9. Statistical Analysis

All statistical data were expressed as the mean ± SD. One Way ANOVA analysis followed by Dunnet’s analysis was performed and a value of *p* < 0.05 was considered significant.

## 5. Conclusions

Excitotoxicity plays an important role in glutamate-induced apoptosis in astrocytes. It was found that morphine could protect astrocytes from glutamate-induced apoptosis through inhibition of glutamate-caused Ca^2+^ overload and ER stress. These results suggested novel evidence for the toxicological mechanism of glutamate and the controversial effect of morphine on CNS cells.
